# Supplementary material for: Modeling glioblastoma heterogeneity as a dynamic network of cell states
Source: Mol Syst Biol. 2021 Sep 16;17(9):e10105. doi: 10.15252/msb.202010105 (PMC8444284; doi:10.15252/msb.202010105)
Supplement: Supplementary file 5 — Source Data for Figure 3 [file MSB-17-e10105-s001.zip › Figure3A_sourcedata/GSEA_3065/hallmarks_state6.GseaPreranked.1623416308702/HALLMARK_MITOTIC_SPINDLE.html]

Details for gene set HALLMARK\_MITOTIC\_SPINDLE[GSEA]

|  || Dataset | state6 |
| Phenotype | NoPhenotypeAvailable |
| Upregulated in class | na\_neg |
| GeneSet | HALLMARK\_MITOTIC\_SPINDLE |
| Enrichment Score (ES) | -0.3259371 |
| Normalized Enrichment Score (NES) | -1.4101336 |
| Nominal p-value | 0.015822785 |
| FDR q-value | 0.071693584 |
| FWER p-Value | 0.649 |
Table: GSEA Results Summary

  

Fig 1: Enrichment plot: HALLMARK\_MITOTIC\_SPINDLE      
 Profile of the Running ES Score & Positions of GeneSet Members on the Rank Ordered List

  

| PROBE | GENE SYMBOL | GENE\_TITLE | RANK IN GENE LIST | RANK METRIC SCORE | RUNNING ES | CORE ENRICHMENT || 1 | WASL |  |  | 188 | 0.180 | -0.0066 | No |
| 2 | ABL1 |  |  | 344 | 0.145 | -0.0123 | No |
| 3 | CDC42 |  |  | 439 | 0.130 | -0.0123 | No |
| 4 | ARHGAP29 |  |  | 453 | 0.128 | -0.0030 | No |
| 5 | WASF2 |  |  | 503 | 0.123 | 0.0017 | No |
| 6 | STAU1 |  |  | 538 | 0.119 | 0.0078 | No |
| 7 | NIN |  |  | 542 | 0.119 | 0.0175 | No |
| 8 | CLASP1 |  |  | 587 | 0.115 | 0.0221 | No |
| 9 | ARF6 |  |  | 593 | 0.115 | 0.0312 | No |
| 10 | MARK4 |  |  | 712 | 0.105 | 0.0263 | No |
| 11 | CLIP2 |  |  | 749 | 0.102 | 0.0308 | No |
| 12 | PALLD |  |  | 752 | 0.102 | 0.0391 | No |
| 13 | SAC3D1 |  |  | 860 | 0.094 | 0.0346 | No |
| 14 | ITSN1 |  |  | 895 | 0.091 | 0.0384 | No |
| 15 | RALBP1 |  |  | 964 | 0.087 | 0.0379 | No |
| 16 | DYNLL2 |  |  | 987 | 0.086 | 0.0426 | No |
| 17 | MARCKS |  |  | 1081 | 0.081 | 0.0387 | No |
| 18 | ARL8A |  |  | 1106 | 0.080 | 0.0427 | No |
| 19 | KIF5B |  |  | 1112 | 0.080 | 0.0489 | No |
| 20 | KIF3C |  |  | 1150 | 0.078 | 0.0512 | No |
| 21 | MYH10 |  |  | 1182 | 0.076 | 0.0540 | No |
| 22 | CDC42EP4 |  |  | 1204 | 0.075 | 0.0579 | No |
| 23 | RANBP9 |  |  | 1242 | 0.074 | 0.0599 | No |
| 24 | MAP1S |  |  | 1330 | 0.070 | 0.0557 | No |
| 25 | ALMS1 |  |  | 1377 | 0.068 | 0.0561 | No |
| 26 | BIN1 |  |  | 1419 | 0.067 | 0.0570 | No |
| 27 | ABI1 |  |  | 1465 | 0.066 | 0.0573 | No |
| 28 | FSCN1 |  |  | 1498 | 0.064 | 0.0590 | No |
| 29 | PPP4R2 |  |  | 1519 | 0.063 | 0.0620 | No |
| 30 | MAP3K11 |  |  | 1576 | 0.061 | 0.0607 | No |
| 31 | PREX1 |  |  | 1790 | 0.054 | 0.0405 | No |
| 32 | ARHGEF3 |  |  | 1883 | 0.051 | 0.0342 | No |
| 33 | STK38L |  |  | 1886 | 0.051 | 0.0382 | No |
| 34 | PXN |  |  | 1948 | 0.049 | 0.0353 | No |
| 35 | CEP250 |  |  | 1954 | 0.049 | 0.0389 | No |
| 36 | RASAL2 |  |  | 1966 | 0.049 | 0.0417 | No |
| 37 | EZR |  |  | 2077 | 0.046 | 0.0328 | No |
| 38 | MID1IP1 |  |  | 2088 | 0.045 | 0.0354 | No |
| 39 | BCR |  |  | 2092 | 0.045 | 0.0389 | No |
| 40 | TSC1 |  |  | 2116 | 0.044 | 0.0400 | No |
| 41 | KIF3B |  |  | 2181 | 0.042 | 0.0361 | No |
| 42 | LATS1 |  |  | 2222 | 0.041 | 0.0349 | No |
| 43 | TLK1 |  |  | 2244 | 0.040 | 0.0358 | No |
| 44 | TTK |  |  | 2262 | 0.040 | 0.0372 | No |
| 45 | CDC42BPA |  |  | 2279 | 0.039 | 0.0386 | No |
| 46 | CD2AP |  |  | 2345 | 0.038 | 0.0343 | No |
| 47 | KATNB1 |  |  | 2380 | 0.036 | 0.0334 | No |
| 48 | EPB41L2 |  |  | 2440 | 0.035 | 0.0295 | No |
| 49 | MAPRE1 |  |  | 2489 | 0.034 | 0.0268 | No |
| 50 | LRPPRC |  |  | 2574 | 0.032 | 0.0197 | No |
| 51 | PCGF5 |  |  | 2586 | 0.031 | 0.0211 | No |
| 52 | UXT |  |  | 2623 | 0.030 | 0.0195 | No |
| 53 | NEDD9 |  |  | 2666 | 0.030 | 0.0171 | No |
| 54 | KATNA1 |  |  | 2681 | 0.029 | 0.0179 | No |
| 55 | ARFGEF1 |  |  | 2682 | 0.029 | 0.0204 | No |
| 56 | ARHGEF7 |  |  | 2777 | 0.027 | 0.0118 | No |
| 57 | NUMA1 |  |  | 3025 | 0.021 | -0.0151 | No |
| 58 | PLK1 |  |  | 3116 | 0.020 | -0.0238 | No |
| 59 | ARHGDIA |  |  | 3321 | 0.015 | -0.0462 | No |
| 60 | ACTN4 |  |  | 3351 | 0.014 | -0.0484 | No |
| 61 | RACGAP1 |  |  | 3432 | 0.012 | -0.0566 | No |
| 62 | RABGAP1 |  |  | 3579 | 0.010 | -0.0727 | No |
| 63 | TRIO |  |  | 3720 | 0.006 | -0.0884 | No |
| 64 | YWHAE |  |  | 3963 | 0.001 | -0.1164 | No |
| 65 | OPHN1 |  |  | 4021 | 0.000 | -0.1230 | No |
| 66 | BCAR1 |  |  | 4073 | -0.001 | -0.1288 | No |
| 67 | TBCD |  |  | 4076 | -0.001 | -0.1290 | No |
| 68 | PAFAH1B1 |  |  | 4151 | -0.002 | -0.1374 | No |
| 69 | CAPZB |  |  | 4179 | -0.003 | -0.1403 | No |
| 70 | SPTBN1 |  |  | 4188 | -0.003 | -0.1410 | No |
| 71 | NF1 |  |  | 4226 | -0.004 | -0.1449 | No |
| 72 | GEMIN4 |  |  | 4232 | -0.004 | -0.1452 | No |
| 73 | FGD4 |  |  | 4236 | -0.004 | -0.1452 | No |
| 74 | KIF1B |  |  | 4348 | -0.006 | -0.1576 | No |
| 75 | RHOT2 |  |  | 4371 | -0.006 | -0.1596 | No |
| 76 | CDC42EP1 |  |  | 4416 | -0.007 | -0.1641 | No |
| 77 | MYH9 |  |  | 4424 | -0.007 | -0.1643 | No |
| 78 | ABR |  |  | 4438 | -0.008 | -0.1652 | No |
| 79 | TIAM1 |  |  | 4472 | -0.008 | -0.1683 | No |
| 80 | ROCK1 |  |  | 4476 | -0.008 | -0.1680 | No |
| 81 | RASA1 |  |  | 4620 | -0.011 | -0.1836 | No |
| 82 | SHROOM2 |  |  | 4660 | -0.012 | -0.1872 | No |
| 83 | FLNB |  |  | 4682 | -0.012 | -0.1886 | No |
| 84 | CCNB2 |  |  | 4736 | -0.013 | -0.1936 | No |
| 85 | TUBGCP5 |  |  | 4745 | -0.013 | -0.1934 | No |
| 86 | WASF1 |  |  | 4764 | -0.014 | -0.1943 | No |
| 87 | NOTCH2 |  |  | 4874 | -0.016 | -0.2056 | No |
| 88 | TAOK2 |  |  | 4892 | -0.016 | -0.2062 | No |
| 89 | ARHGAP10 |  |  | 5016 | -0.019 | -0.2189 | No |
| 90 | TUBGCP6 |  |  | 5034 | -0.019 | -0.2193 | No |
| 91 | EPB41 |  |  | 5038 | -0.019 | -0.2180 | No |
| 92 | SPTAN1 |  |  | 5102 | -0.020 | -0.2236 | No |
| 93 | RASA2 |  |  | 5246 | -0.023 | -0.2382 | No |
| 94 | ARFIP2 |  |  | 5251 | -0.023 | -0.2367 | No |
| 95 | APC |  |  | 5342 | -0.025 | -0.2450 | No |
| 96 | BUB1 |  |  | 5377 | -0.026 | -0.2468 | No |
| 97 | NCK1 |  |  | 5409 | -0.027 | -0.2481 | No |
| 98 | SASS6 |  |  | 5420 | -0.027 | -0.2471 | No |
| 99 | ARHGEF12 |  |  | 5476 | -0.028 | -0.2511 | No |
| 100 | CEP131 |  |  | 5480 | -0.028 | -0.2491 | No |
| 101 | ARHGEF2 |  |  | 5504 | -0.029 | -0.2493 | No |
| 102 | ALS2 |  |  | 5539 | -0.029 | -0.2508 | No |
| 103 | LMNB1 |  |  | 5545 | -0.029 | -0.2489 | No |
| 104 | ECT2 |  |  | 5560 | -0.030 | -0.2480 | No |
| 105 | SUN2 |  |  | 5620 | -0.031 | -0.2522 | No |
| 106 | PLEKHG2 |  |  | 5823 | -0.035 | -0.2727 | No |
| 107 | RAB3GAP1 |  |  | 5876 | -0.036 | -0.2757 | No |
| 108 | ARHGEF11 |  |  | 5899 | -0.036 | -0.2753 | No |
| 109 | TUBGCP2 |  |  | 5982 | -0.038 | -0.2816 | No |
| 110 | CCDC88A |  |  | 6086 | -0.040 | -0.2902 | No |
| 111 | PCNT |  |  | 6116 | -0.041 | -0.2901 | No |
| 112 | CYTH2 |  |  | 6138 | -0.041 | -0.2891 | No |
| 113 | MID1 |  |  | 6187 | -0.042 | -0.2911 | No |
| 114 | FARP1 |  |  | 6319 | -0.045 | -0.3025 | No |
| 115 | HOOK3 |  |  | 6406 | -0.047 | -0.3084 | No |
| 116 | CSNK1D |  |  | 6416 | -0.048 | -0.3055 | No |
| 117 | CEP57 |  |  | 6420 | -0.048 | -0.3018 | No |
| 118 | ATG4B |  |  | 6464 | -0.049 | -0.3027 | No |
| 119 | PDLIM5 |  |  | 6585 | -0.052 | -0.3122 | No |
| 120 | NCK2 |  |  | 6607 | -0.053 | -0.3101 | No |
| 121 | SSH2 |  |  | 6632 | -0.054 | -0.3084 | No |
| 122 | KIFAP3 |  |  | 6776 | -0.058 | -0.3200 | Yes |
| 123 | CDC27 |  |  | 6787 | -0.058 | -0.3163 | Yes |
| 124 | HDAC6 |  |  | 6848 | -0.060 | -0.3182 | Yes |
| 125 | SOS1 |  |  | 6849 | -0.060 | -0.3131 | Yes |
| 126 | CENPJ |  |  | 6863 | -0.061 | -0.3095 | Yes |
| 127 | PRC1 |  |  | 6918 | -0.062 | -0.3105 | Yes |
| 128 | TUBGCP3 |  |  | 6972 | -0.064 | -0.3112 | Yes |
| 129 | BIRC5 |  |  | 7024 | -0.066 | -0.3116 | Yes |
| 130 | SMC3 |  |  | 7087 | -0.067 | -0.3131 | Yes |
| 131 | KIF22 |  |  | 7098 | -0.068 | -0.3085 | Yes |
| 132 | CLIP1 |  |  | 7187 | -0.071 | -0.3128 | Yes |
| 133 | CDK5RAP2 |  |  | 7213 | -0.071 | -0.3097 | Yes |
| 134 | RICTOR |  |  | 7252 | -0.073 | -0.3079 | Yes |
| 135 | AKAP13 |  |  | 7351 | -0.076 | -0.3129 | Yes |
| 136 | PKD2 |  |  | 7365 | -0.077 | -0.3079 | Yes |
| 137 | TOP2A |  |  | 7430 | -0.079 | -0.3087 | Yes |
| 138 | CEP192 |  |  | 7515 | -0.082 | -0.3115 | Yes |
| 139 | KLC1 |  |  | 7558 | -0.084 | -0.3093 | Yes |
| 140 | KIF11 |  |  | 7619 | -0.087 | -0.3089 | Yes |
| 141 | AURKA |  |  | 7700 | -0.091 | -0.3105 | Yes |
| 142 | NDC80 |  |  | 7734 | -0.093 | -0.3065 | Yes |
| 143 | BRCA2 |  |  | 7772 | -0.094 | -0.3029 | Yes |
| 144 | SORBS2 |  |  | 7917 | -0.102 | -0.3110 | Yes |
| 145 | SMC1A |  |  | 8047 | -0.110 | -0.3167 | Yes |
| 146 | CTTN |  |  | 8073 | -0.112 | -0.3101 | Yes |
| 147 | DLG1 |  |  | 8081 | -0.112 | -0.3015 | Yes |
| 148 | VCL |  |  | 8092 | -0.113 | -0.2931 | Yes |
| 149 | FLNA |  |  | 8171 | -0.119 | -0.2921 | Yes |
| 150 | ANLN |  |  | 8181 | -0.120 | -0.2831 | Yes |
| 151 | MYO9B |  |  | 8203 | -0.122 | -0.2752 | Yes |
| 152 | RFC1 |  |  | 8205 | -0.122 | -0.2651 | Yes |
| 153 | KIF15 |  |  | 8228 | -0.124 | -0.2572 | Yes |
| 154 | CNTRL |  |  | 8234 | -0.124 | -0.2473 | Yes |
| 155 | KIF4A |  |  | 8300 | -0.132 | -0.2437 | Yes |
| 156 | FBXO5 |  |  | 8310 | -0.133 | -0.2335 | Yes |
| 157 | KIF2C |  |  | 8328 | -0.135 | -0.2240 | Yes |
| 158 | GSN |  |  | 8339 | -0.137 | -0.2136 | Yes |
| 159 | CDK1 |  |  | 8351 | -0.139 | -0.2032 | Yes |
| 160 | TPX2 |  |  | 8370 | -0.143 | -0.1932 | Yes |
| 161 | KNTC1 |  |  | 8395 | -0.146 | -0.1837 | Yes |
| 162 | DLGAP5 |  |  | 8447 | -0.152 | -0.1768 | Yes |
| 163 | KIF23 |  |  | 8465 | -0.154 | -0.1657 | Yes |
| 164 | ARHGAP5 |  |  | 8494 | -0.160 | -0.1555 | Yes |
| 165 | PCM1 |  |  | 8496 | -0.160 | -0.1421 | Yes |
| 166 | NUSAP1 |  |  | 8523 | -0.167 | -0.1310 | Yes |
| 167 | CKAP5 |  |  | 8532 | -0.169 | -0.1177 | Yes |
| 168 | KIF20B |  |  | 8576 | -0.180 | -0.1075 | Yes |
| 169 | DST |  |  | 8644 | -0.211 | -0.0974 | Yes |
| 170 | PIF1 |  |  | 8696 | -0.238 | -0.0833 | Yes |
| 171 | CENPF |  |  | 8703 | -0.240 | -0.0637 | Yes |
| 172 | CENPE |  |  | 8734 | -0.270 | -0.0444 | Yes |
| 173 | DYNC1H1 |  |  | 8761 | -0.302 | -0.0220 | Yes |
| 174 | SMC4 |  |  | 8764 | -0.307 | 0.0037 | Yes |
Table: GSEA details [plain text format]

  

Fig 2: HALLMARK\_MITOTIC\_SPINDLE: Random ES distribution      
 Gene set null distribution of ES for **HALLMARK\_MITOTIC\_SPINDLE**
